# *Echinococcus granulosus* promotes MAPK pathway-mediated osteoclast differentiation by inhibiting Nrf2 in osseous echinococcosis

**DOI:** 10.1186/s13567-025-01510-2

**Published:** 2025-04-12

**Authors:** Yaqing Liu, Jing Li, Zhendong Zhang, Qi Li, Yanhu Tian, Sibo Wang, Chenhui Shi, Haohao Sun

**Affiliations:** 1 The First Affiliated Hospital of Shihezi University, Xinjiang Uygur Autonomous Region, Shihezi, 832000 China; 2 The Medical College of Shihezi University, Xinjiang Uygur Autonomous Region, Shihezi, 832000 China; 3 Xi’an Jiaotong University Affiliated HongHui Hospital, Beilin district, Xi’an, 710000 Shanxi China

**Keywords:** *Echinococcus granulosus*, osseous cystic echinococcosis, Nrf2, osteoclasts

## Abstract

Osseous echinococcosis causes severe “osteolytic” changes in the bone tissue of *Echinococcus granulosus* (*E. granulosus*) infection sites by promoting the over-differentiation of osteoclasts at the site. Nrf2 is a key regulator of osteoclast differentiation and formation, and this study investigated the regulatory mechanism by which Nrf2 promotes osteoclast differentiation after *E. granulosus* infection. In vitro, our study revealed that PSC intervention suppressed the expression levels of intracellular Nrf2 and its downstream effector, heme oxygenase-1 (HO-1), while increasing the content of intracellular reactive oxygen species (ROS), thereby promoting osteoclast differentiation. Next, we treated bone marrow mononuclear cells (BMMCs) with protoscoleces (PSC) and found that Nrf2 knockdown significantly promoted osteoclast formation, whereas Nrf2 activation had the opposite effect. We also verified that phosphorylation of the MAPK pathway was promoted after PSC intervention. In vivo, we established an osseous CE model and reported that Nrf2 knockout mice presented more pronounced bone destruction and more active osteoclast differentiation in infected bone tissue. In this study, we demonstrated that Nrf2 plays an important regulatory role in echinococcosis of the bone caused by *E. granulosus* infection both in vitro and in vivo. *E. granulosus* infection inhibits the expression of Nrf2 in cells, which leads to increased osteoclast differentiation and active bone resorption. This study provides not only a direction for more precise mechanistic research but also a new molecular target for the drug treatment of osseous echinococcosis.

## Introduction

Cystic echinococcosis (CE) is a global zoonotic disease prevalent in pastoral areas. It is the most common form of echinococcosis caused by infection with *Echinococcus granulosus* (*E. granulosus*) [1]. Infected foci often involve multiple organs throughout the body, such as the liver (70%) and lungs (20%), whereas osseous CE accounts for 0.5–4% of all cases of CE [2, 3]. In contrast to more established clinical treatments for liver CE, treatment for osseous CE is relatively unitary and is characterized by high rates of recurrence, disability, and mortality [4, 5]. Moreover, during clinical diagnosis and treatment, osseous CE shows a pronounced “osteolytic” phenomenon, wherein bone tissues at the infected site are destroyed in large quantities, eventually leading to pathological fractures in patients [6]. In our previous study, we demonstrated that *E. granulosus* infection promoted osteoclast differentiation and bone destruction at the infection site [7]. Current studies have shown that *E. granulosus* infection can induce an increase in ROS in organisms [8], and the role of the Nrf2/ROS axis in the regulation of PSC activity has been widely studied [9]. Therefore, we used nuclear factor erythroid 2-related factor 2 (Nrf2) as a breakthrough point in our study to investigate the regulatory role of Nrf2 in the promotion of osteoclast differentiation by PSC.

Nrf2 is derived from the basic leucine zipper protein family, which has been confirmed in various cells, including osteoblasts and osteoclasts, where it serves as a major regulator of intracellular oxidative stress processes [10]. Targeted activation of Nrf2 has shown potential efficacy in the treatment of oxidative and inflammatory diseases [11]. PtAu2 nanomaterials synthesized by Chen et al. can deregulate the binding of Keap1 to Nrf2 in the cytoplasm and promote Nrf2 translocation to upregulate the expression of endogenous anti-inflammatory and antioxidant products, effectively inhibiting inflammatory osteolysis [12]. Bitopertin [13], an Nrf2 agonist drug used clinically for the treatment of psychiatric disorders, has been found to have excellent antioxidant effects [14]. Yao et al. reported another Nrf2 agonist, SFN, which significantly downregulated intracellular ROS levels by promoting the nuclear translocation of Nrf2 in RAW264.7 cells. This effect inhibited RANKL-induced osteoclast differentiation in vitro and inhibited LPS-induced inflammatory bone loss in vivo [15]. Analogues or agonists of Nrf2 have been shown to have antiosteoporotic potential and are potential candidates for inhibiting osteoclast over differentiation [16].

In our previous studies, we showed that the osteolytic manifestation of osseous CE is related to increased osteoclast differentiation at the infection site and active bone resorption [7]. However, the specific regulatory mechanism remains unclear. Although the regulatory role of Nrf2 has been studied in bone metabolic diseases, such as osteoporosis, its involvement in regulating osteoclast differentiation in osseous CE following *E. granulosus* infection has not been investigated. In this study, we aimed to identify and elucidate the regulatory role of Nrf2 in osseous CE to investigate the mechanism of the “osteolysis” phenomenon and facilitate the development of molecularly targeted drugs for treating osseous CE in clinical settings.

## Materials and methods

### Ethical approval

Eight- to ten-week-old C57BL/6 mice were purchased from Wuhan Solarbio Biotechnology Co., Ltd. Nrf2^−/−^ mice, generated via CRISPR-Cas9 technology, were a gift from Professor Feng Li (Orthopedic Centre, Tongji Medical College, Huazhong University of Science and Technology, China). Animal experiments were designed and conducted with approval from the Medical Ethics Committee of the First Affiliated Hospital of Shihezi University (Ethics Approval No. A2022-203–01).

### Antibodies and reagents

The target antibodies against c-Fos (#ab190289, 1:1000), tartrate-resistant acid phosphatase (TRAP, #ab191406, 1:1000), NFATc1 (#ab2722, 0.5 μg/mL), CathK (#ab19027, 1:2000), Nrf2 (#ab62352, 1:1000), HO-1 (#ab189491, 1:1000), and β-actin (#ab8226, 1:5000) were purchased from Abcam (Cambridge, UK). MAPK pathway-related antibodies against p38 (#8690 T, 1:1000), p-p38 (#4511 T, 1:1000), ERK (#4695 T, 1:1000), p-ERK (#4370 T, 1:1000), JNK (#9252 T, 1:1000), and p-JNK (#4668 T, 1:1000) were acquired from Cell Signaling Technology (Massachusetts, USA). An F-actin staining kit (#ab112125) and a ROS content detection kit (#ab113851) were purchased from Abcam. TRAP staining kits were obtained from Sigma‒Aldrich (St. Louis, MO, USA). Recombinant mouse M-CSF (#416-ML/CF) and recombinant mouse RANKL (#462-TEC) were obtained from R&D Systems (Minnesota, USA). The lentiviruses packaged with siNrf2 and scrambled controls were purchased from Genechem Technology (Shanghai, China).

### Extraction and culture of protoscoleces of *E. granulosus*

Protoscoleces were purchased from the Changji City Livestock Slaughterhouse, and the specific extraction method and activity assay used were described in our previous study [7]. Following the complete extraction of the original head nodes, they were incubated at 37 °C and 5% CO_2_ in 1640 complete medium containing 10% fetal bovine serum.

### Osteoclast-induced differentiation

C57BL/6 mice aged 4–6 weeks were selected for euthanasia and transferred to an ultraclean table to retain their femurs and tibiae. Both ends of the femur and tibia were cut off with sterile scissors, and sterile PBS was extracted with a 5 mL syringe and inserted into the broken end of the diaphysial shaft to rinse the medullary cavity repeatedly until it turned white. The PBS suspension containing the contents of the medullary lumen was collected, centrifuged and then lysed with erythrocyte lysate at 0 °C for 5 min, followed by centrifugation to remove the supernatant and retain the extracted cells. The extracted cells were cultured in α-MEM complete medium containing 30 ng/mL M-CSF for 16 h. The supernatant of the culture mixture was then collected and centrifuged. BMMCs were obtained after discarding the supernatant and were subjected to cell counting. The cells were then inoculated into 96-well plates for osteoclast induction and differentiation at a concentration of 1.5 × 10^4^ cells per well and cultured in complete medium containing 30 ng/mL M-CSF and 100 ng/mL RANKL. The culture conditions used during induced differentiation were as follows: temperature, 37 °C; CO_2_ concentration, 5%; and half-volume fluid was changed every two days for 5‒7 days.

### TRAP staining and F-actin ring staining

Osteoclasts were washed twice with PBS, fixed with 5% paraformaldehyde, and then washed with deionized distilled water. TRAP staining solution was used to stain the osteoclasts. These cells were then observed as multinucleated cells with a purplish-red color under a microscope. After 5% paraformaldehyde fixation and permeabilization of the cells with 0.1% Triton X-100, the cells were incubated with phalloidin working solution for 40 min at 37 °C in the dark. The F-actin ring was observed as a green ring under a fluorescence microscope.

### Cell viability

BMMCs were inoculated into 96-well plates at a density of 1 × 10^4^ cells per well and cultured in complete medium for 48 h. After the medium was changed, cell culture was initiated with complete medium containing 30 ng/mL M-CSF, and different amounts of PSCs (0, 5, 10, 20, 40, 60, 80, and 100/100 µL complete medium) were added simultaneously. The medium was changed after 12, 24, and 36 h of coculture. Next, 10 µL of CCK-8 solution and 90 µL of complete medium were added to each well and incubated for 3 h in the cell culture incubator, followed by determination of cell viability via an enzyme marker (Thermo Fisher Scientific, MA, USA) at 450 nm. Cell viability was calculated as a percentage of the OD value of the PSC intervention group to the OD value of the control group.

### Detection of intracellular ROS levels

The cells were washed twice with PBS before staining, and the intracellular ROS levels were detected via the fluorescent probe dichlorofluorescein diacetate. Staining was performed in an incubator for 2 h, after which the cells were washed twice. The ROS levels were quantified using fluorescence microscopy under excitation light at 540 nm, followed by quantitative analysis via ImageJ software.

### SiRNA transfection

BMMCs were cultured on 60-mm plates (4 × 10^5^ cells per plate), and α-MEM containing 10% BSA (Gibco, USA) and antibiotics (100 mg/mL streptomycin and 100 units/mL penicillin, Gibco, USA) was added 12 h later to prepare for transfection. A lentivirus-based siRNA expression system was used to knock down Nrf2 gene expression. After 48 h of infection with siRNAs, the BMMCs were washed with sterile PBS, and the medium was replaced with α-MEM containing 10% BSA, antibiotics, and 30 ng/mL M-CSF and incubated at 37 °C for 16 h. After incubation, the BMMCs were subjected to osteoclast induction and differentiation or detected by western blotting for the expression of intracellular proteins in the transfected cells. Nrf2 (1:1000) intracellular protein expression was detected by western blotting to clarify the efficiency of siRNA transfection.

### Real-time quantitative PCR

Total RNA was isolated from primary cells cultured in osteoclast induction medium for 24 h using an RNA extraction kit (#RC112-01; Vazyme Biotech, Nanjing, China). The complementary DNA (cDNA) was generated using a reverse transcription kit (#11141ES60, Yeasen). Real-time PCR amplification was then performed using the prepared cDNA. The following primer sequences were used: c-Fos: forward 5′-CCAGTCAAGAGCATCAGCAA-3′ and reverse 5′-AAGTAGTGCAGCCCGGAGTA-3′; TRAP: forward 5′-TACCTGTGTGGACATGACC-3′ and reverse 5′-CAGATCCATAGTGAAACCGC-3′; NFATc1: forward 5′-GGTCTTCCGAGTTCACATCC-3′ and reverse 5′-CACAGGTCCCGGTCAGTC-3′; CTSK: forward 5′-TGGACTATACCCAGGGAAACCTC-3′ and reverse 5′-CAAGTAACTATGATGCCCAAGCAG-3′; Nrf2: forward AGGTTGCCCACATTCCCAAA and reverse GGGAATGTCTGCGCCAAAAG; β-actin: forward 5′-GTCGTACCACAGGCATTGTGATGG-3′ and reverse 5′-GCAATGCCTGGGTACATGGTGG-3′. The assay was performed according to the amplification kit (#CW0957M, YEASEN, Hangzhou, China) instructions. Three replicates were prepared for each sample, and the relative mRNA levels of the target genes were calculated using the 2^−ΔΔCT^ method, with β-actin as the internal control.

### Western blotting

Total protein from cultured cells was extracted using RIPA lysis buffer containing 1% protein phosphatase inhibitor, and the protein concentration was determined using a bicinchoninic acid kit (Boster Biotechnology, Wuhan, China). Protein gel electrophoresis was performed using 8–12% sodium dodecyl sulfate‒polyacrylamide gels at a constant voltage of 90 V. The proteins were transferred to polyvinylidene difluoride (PVDF) membranes (Millipore, Boston, USA) and incubated at room temperature with Tris-buffered saline/Tween-20 (TBST) containing 5% bovine serum albumin. The membranes were then incubated with antibodies against the target proteins for 12–16 h. The membranes were washed three times with TBST and further incubated with horseradish peroxidase-coupled secondary antibodies for 2 h. Finally, the protein levels were detected using an enhanced chemiluminescence reagent, with β-actin serving as an internal control to normalize the target protein.

### Establishment of a mouse model of osseous CE

C57BL/6 mice aged 8–10 weeks were selected for the generation of the femoral CE disease model. Sevoflurane inhalation was used to anaesthetize the mice, setting the initial induction flow rate of sevoflurane to 4%, maintaining the flow rate at 2% intraoperatively until the end of the operation (the operation could be performed if there was no spontaneous body-motion response to the pain stimulus given to the mice and if the operation time did not exceed 30 min). First, the hair in the knee joint area of each mouse was shaved and sterilized with iodophor. In the experimental group, a 1 mL syringe was used to extract the suspension of culture medium containing the PSC, the needle was punctured from the knee joint, and 0.3 mL of PSC suspension (1 × 10^4^ PSC/mL) was injected subperiosteum against the periosteum. In the control group, 0.3 mL of saline was injected subperiosteally via the same route. Thirty mice were randomly selected from the experimental and control groups, and both groups were euthanized for subsequent experiments after 6 months of normal feeding under the same feeding conditions at the end of modelling.

### Micro- computed tomography (CT) analyses

Following a 6-month modelling period, the mice were euthanized via decapitation. The femurs were harvested from both sides and subsequently fixed in 4% paraformaldehyde. The right femur was analysed histomorphometrically using a micro-CT system (Scanco Medical) in a uniform manner. The scanning parameters were set to 100 kV, 98 mA, and 10 pixels/pixel. Finally, the built-in software of micro-CT was used to analyse the bone parameters and perform three-dimensional reconstruction.

### Immunohistochemical staining

Stripped femurs were decalcified using an ethylenediaminetetraacetic acid (EDTA, pH 7.5) decalcification solution for 1 month, paraffin-embedded, and finally cut into 4 μm thick tissue sections. The tissue sections were subjected to xylene dewaxing and hydration, antigen retrieval, and blocking of endogenous peroxidase activity. The samples were then incubated with Nrf2 antibody (1:400) overnight at 4 ℃. The samples were subsequently incubated overnight at 4 °C with the appropriate concentration of antibody. The next day, the sections were transferred to an incubator at 37 ℃ for 45 min for rewarming. Then, the HRP-conjugated secondary antibody was added dropwise, and the mixture was incubated at 20 ℃ for 30 min. Finally, the mixture was incubated with 3,3′-diaminobenzidine (DAB) for 3 min before hematoxylin staining. The samples were then dehydrated, sealed, and imaged under a microscopic imaging system (E600, Eclipse, Nikon, Japan).

### Histomorphological staining

The tissue sections were dewaxed and hydrated and then stained with hematoxylin and eosin (H&E). TRAP staining was performed by immersing the sections in TRAP working solution, after which the sections were sealed with neutral gum before being observed under a microscope (E600, Eclipse, Nikon, Japan) for analysis. Under the microscope, the purple‒red colour of the osteoclasts was observed. Finally, ImageJ software was used to calculate the percentage of the positive area of osteoclasts in the total bone tissue area.

### Quantitation and statistical analysis

All data from this study were statistically analysed and plotted with GraphPad Prism 8.0 software, with at least three independent replicates for each experiment. The data are presented as the means ± standard deviations. Comparisons between two groups were performed using an independent samples *t*-test, and comparisons between multiple groups were performed using one-way analysis of variance (ANOVA). Statistical significance is indicated by *P < 0.05, **P < 0.01, ***P < 0.001, and ****P < 0.0001.

## Results

### PSC of *Echinococcus granulosus* promotes osteoclast differentiation

**Figure 1:**
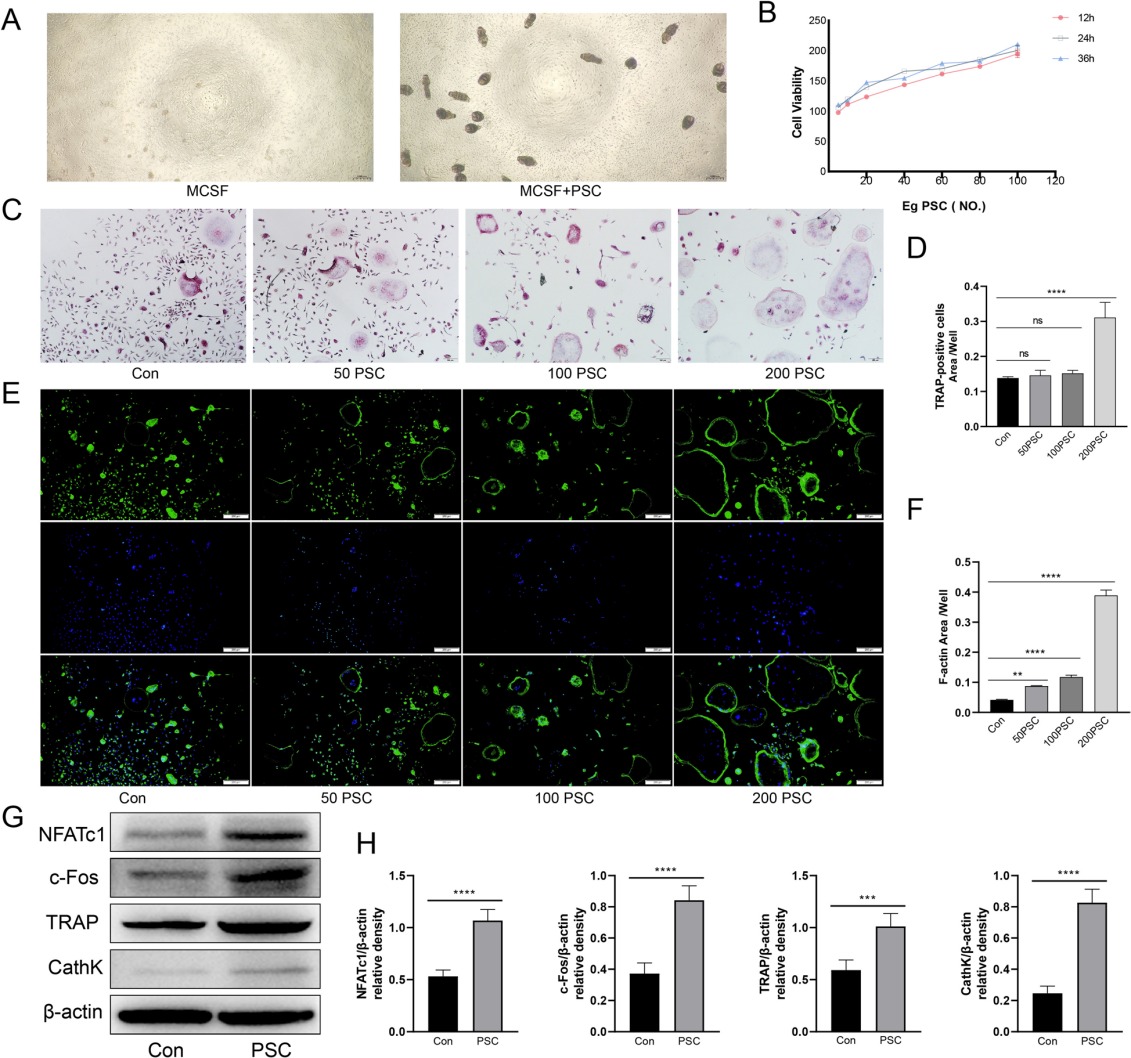
**PSC increases osteoclast viability and promote osteoclast maturation and differentiation.** **A** The effect on BMMCs was observed by adding PSC to complete medium containing M-CSF. **B** A CCK-8 assay was performed to detect the effect of PSC on BMMC activity at 12, 24, and 36 h. **C**, **D** BMMCs were grouped for induced differentiation under different interventions, and osteoclast formation was detected using TRAP. Four groups were formed: the control, low-dose PSC (50/well), medium-dose PSC (100/well), and high-dose PSC (200/well) groups. **E**, **F** Phalloidin fluorescence staining was used to detect F-actin ring formation in the same subgroups. **G**, **H** Western blotting was used to detect the expression of osteoclast-related proteins after PSC intervention. *P < 0.05, **P < 0.01, ***P < 0.001, ****P < 0.0001. Three replicates were used for each dataset.

To investigate the effect of PSC on the differentiation of osteoclast precursor cells into osteoclasts, we established a coculture system of PSC and BMMCs. This coculture did not lead to changes in the morphology of BMMCs compared with that of the control group. Similarly, the activity of the PSC itself did not change significantly. Under the microscope, the PSC appeared rounded and translucent and exhibited slow peristaltic movements (Figure 1A). The results of the CCK-8 assay revealed that when PSC were cocultured with BMMCs, they promoted BMMC activity (Figure 1B). TRAP staining after 5 days of coculture with osteoclast-inducing medium revealed an increase in the number of TRAP-positive cells and stronger differentiation ability with the addition of PSC than in the control group (Figures 1C and D). Phalloidin staining revealed that PSC intervention promoted the formation of the F-actin ring, yielding results that were statistically comparable to those of TRAP staining (Figures 1E and F). Western blot analysis further demonstrated that PSC intervention promoted the expression of osteoclast-related proteins (Figures 1G and H).

### *Echinococcus granulosus* inhibits Nrf2 expression during osteoclast differentiation

**Figure 2:**
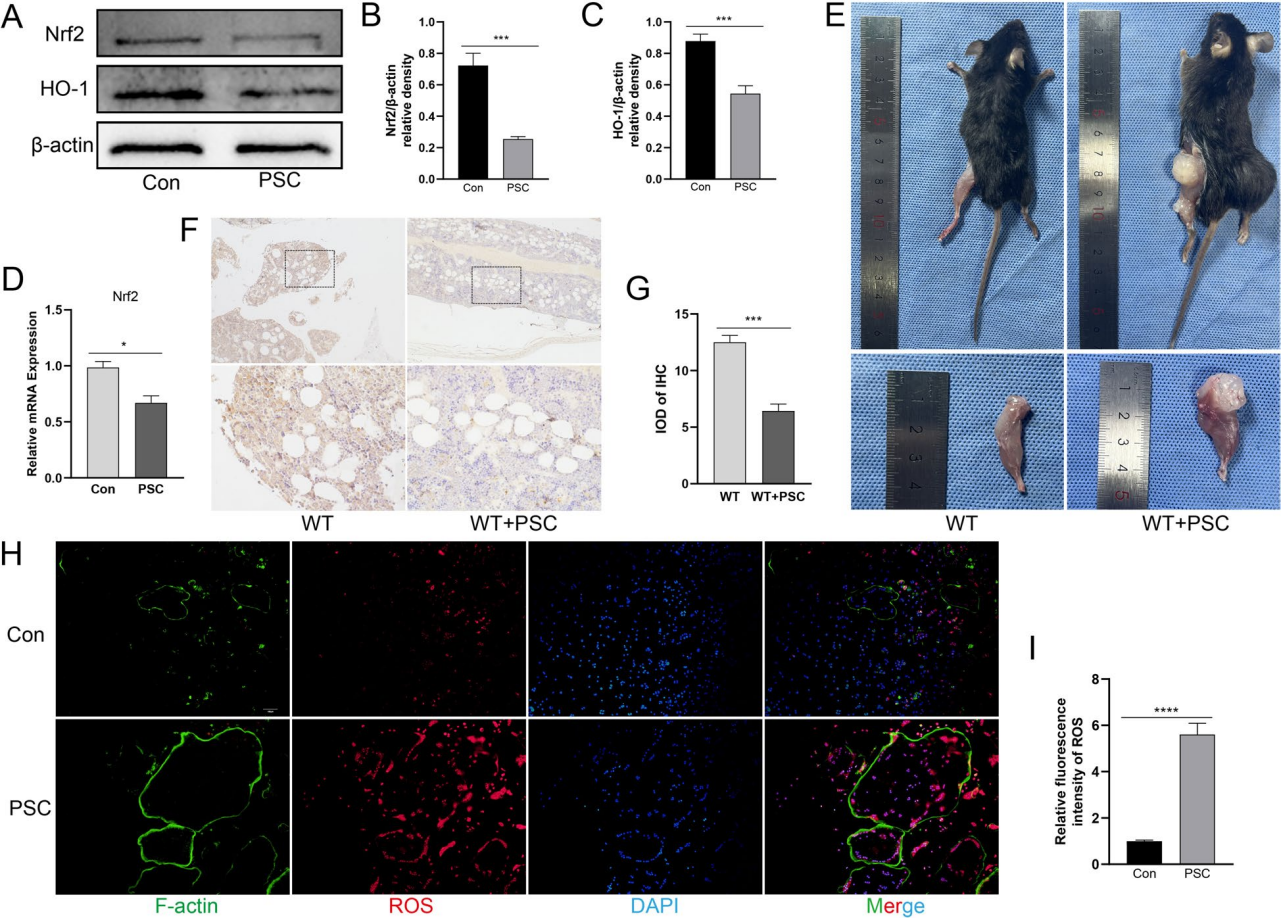
**PSC inhibits Nrf2 expression during osteoclast differentiation**. **A**–**C** Protein expression of intracellular Nrf2 and HO-1 after PSC stimulation. **D** Nrf2 mRNA gene expression after PSC intervention. **E** Cyst formation visible in isolated femoral tissues from the mouse model. **F**, **G** Immunohistochemical (IHC) staining of isolated femoral tissues. **H**, **I** Fluorescence staining was used to detect intracellular ROS levels in BMMCs. The black dotted boxes represent amplified sections. *P < 0.05, **P < 0.01, ***P < 0.001, ****P < 0.0001.

The expression of the antioxidant-related factor Nrf2 was analysed after PSC intervention. We found that the expression of Nrf2 in BMMCs was significantly suppressed following PSC intervention, along with that of HO-1, an effector regulated downstream of Nrf2 (Figure  2A–C). PCR analysis confirmed these findings (Figure 2D). We subsequently constructed a mouse femoral echinococcosis model and performed immunohistochemical staining of the infected bone tissues, which also revealed a decrease in the Nrf2 protein (Figures 2E–G). ROS, indispensable signaling factors in cellular oxidative activity, play important regulatory roles in osteoclast differentiation [17]. Further studies revealed an increase in intracellular ROS levels after PSC intervention, which was positively correlated with osteoclast formation (Figures 2H, I). These results suggest that PSC intervention inhibits the expression of the antioxidant factor Nrf2, leading to increased intracellular ROS levels, thereby promoting osteoclast differentiation.

### Nrf2 gene deficiency promotes osteoclast differentiation in response to PSC intervention

**Figure 3:**
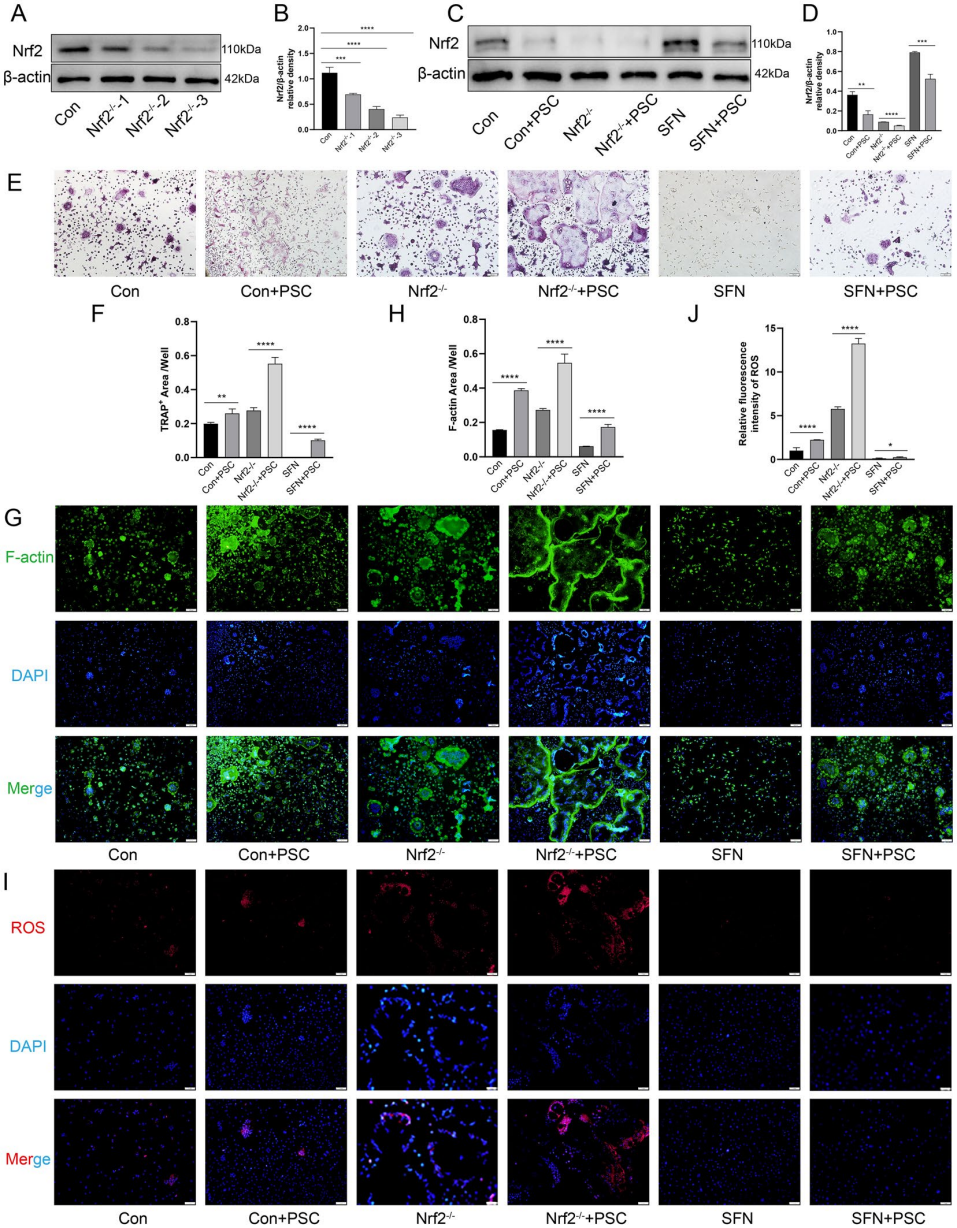
**PSC regulates osteoclast differentiation through Nrf2 expression.** **A**, **B** Transfection efficiency was determined by western blotting after three lentiviral transfections in BMMCs. **C**, **D** Nrf2 protein expression was detected after BMMCs were subjected to different interventions. Six groups were formed: normal induction, PSC induction, Nrf2 silencing, Nrf2 silencing with PSC induction, SFN, and SFN with PSC induction. **E**, **F** BMMCs were grouped for induced differentiation under different interventions, and osteoclast formation was detected using TRAP. **G**, **H** Fluorescence staining with phalloidin was used to detect the formation of the F-actin ring. **I**, **J** Fluorescence staining was used to detect ROS expression in osteoclasts. *P < 0.05, **P < 0.01, ***P < 0.001, ****P < 0.0001.

To verify the regulatory role of Nrf2 after PSC intervention, we first used siNrf2 lentivirus to infect BMMCs, as verified by western blotting. The highest transfection efficiency was observed for siNrf2-3 (Figures 3A and B), and sulforaphane (SFN) was selected as an Nrf2 agonist. BMMCs were divided into control, Nrf2^−/−^, and SFN groups and induced with 100 ng/mL RANKL, with or without the addition of PSC, to examine the differentiation and formation of osteoclasts after PSC intervention at different Nrf2 expression levels (Figures 3C and D). TRAP staining revealed that the addition of different levels of Nrf2 promoted osteoclast formation in PSC compared with that in the control group. Osteoclast formation was greatest after the addition of PSC with Nrf2 silencing. Although SFN inhibited the formation of osteoclasts, this inhibition was partially reversed after the addition of PSC (Figure 3E). Statistical analysis of the osteoclast-positive areas revealed consistent outcomes (Figure 3F). The F-actin ring is a specialized extracellular structure on the surface of osteoclasts; therefore, detecting its formation helps illustrate changes in osteoclast function. The fluorescence staining results revealed that F-actin ring formation was significantly increased after Nrf2 silencing but was inhibited after SFN intervention. However, the addition of PSC promoted F-actin ring formation under various conditions (Figures 3G and H). These findings suggest that PSC promote osteoclast differentiation and their bone resorption function by downregulating Nrf2. Finally, fluorescence detection revealed that ROS levels in osteoclasts increased in response to the addition of PSC under different conditions, and this trend was positively correlated with osteoclast differentiation (Figures 3I and J). Collectively, these results demonstrate that PSC intervention during the induced differentiation of BMMCs into osteoclasts results in the downregulation of the Nrf2 gene and an increase in ROS, which in turn promotes osteoclast differentiation and function.

### Nrf2 knockdown promotes osteoclast-related gene and protein expression under PSC intervention

**Figure 4:**
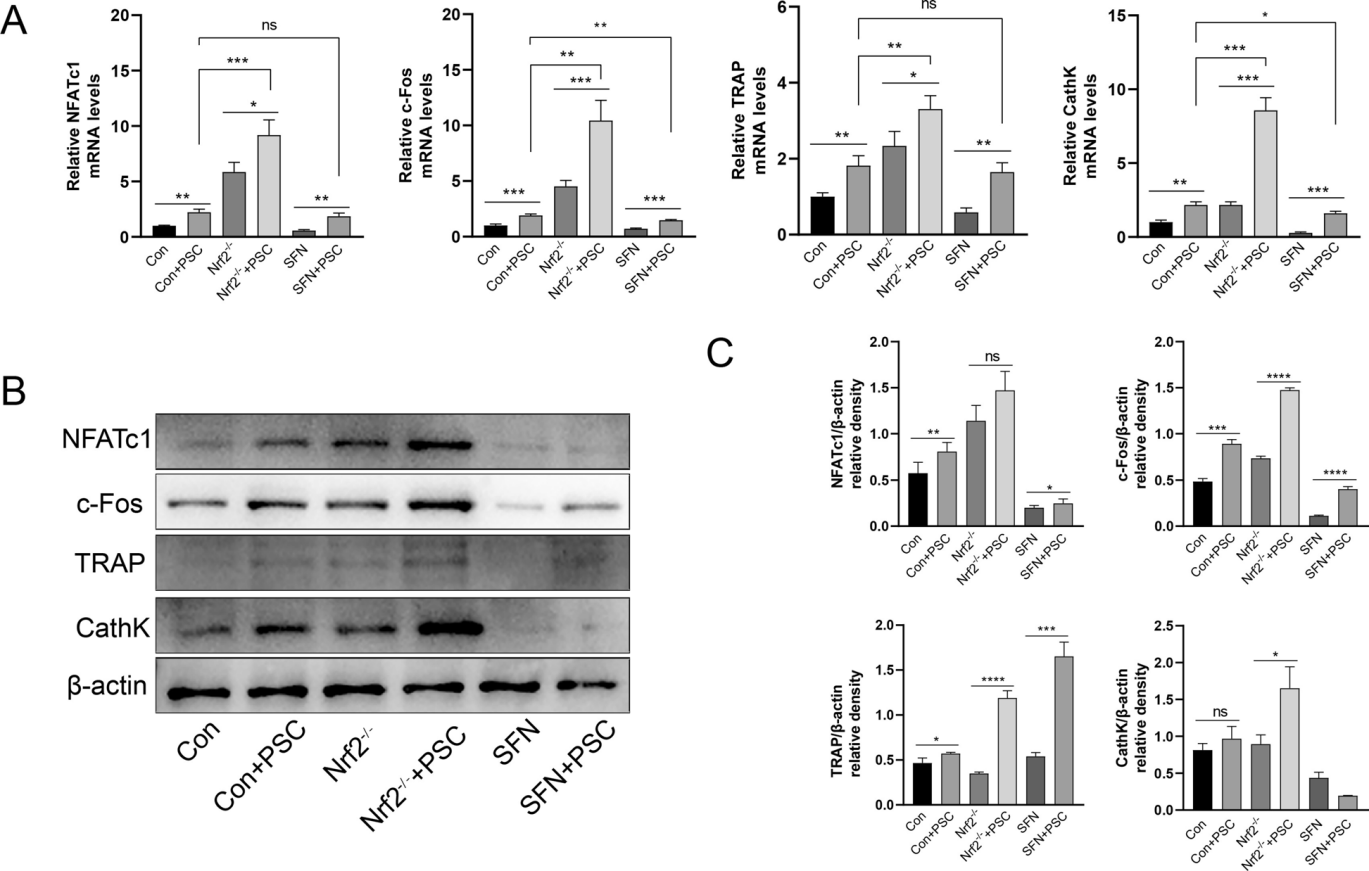
**PSC regulates osteoclast-related gene and protein expression through high and low expression of Nrf2**. **A** BMMCs were cultured and grouped as follows: normal induction, PSC induction, Nrf2 silencing, Nrf2 silencing with PSC induction, SFN, and SFN with PSC induction. Total RNA was extracted after 24 h of culture, after which the target genes were detected. **B**, **C** The same groups were cultured and induced for 3 days, and total proteins were extracted and analysed by western blotting for the detection of target proteins. *P < 0.05, **P < 0.01, ***P < 0.001.

The TRAP and F-actin staining results confirmed that Nrf2 was involved in the regulation of osteoclast differentiation and function after PSC intervention. However, its effect on osteoclast-related genes and proteins requires further verification via the same groupings as those used in previous experiments. RT‒qPCR assays revealed that the transcription of osteoclast-related genes (NFATc1, c-Fos, TRAP, and CathK) was promoted after PSC intervention across different Nrf2 expression levels. The most significant increase in transcription levels occurred when Nrf2 was inhibited and PSC intervention was increased. When an Nrf2 agonist was added, the transcription levels of these genes were greater than those in the groups receiving the Nrf2 agonist and PSC intervention. The transcription of genes repressed by SFN was somewhat reversed in the agonist group compared with the Nrf2 agonist and PSC intervention groups (Figure 4A). Western blotting was used to examine the expression of osteoclast-associated proteins (NFATc1, c-Fos, TRAP, and CathK), and the results revealed that the changes in NFATc1, c-fos and TRAP expression were consistent with the corresponding changes in mRNA expression (Figures 4B and C). Moreover, this promotion was further enhanced after the inhibition of Nrf2. While the agonistic expression of Nrf2 inhibited the expression of these genes and proteins, PSC intervention reversed this inhibition to a certain extent. However, the results of the protein assay of CathK were somewhat different from its mRNA expression trend, and the western blotting results revealed that CathK expression was inhibited after the addition of SFN, but the addition of PSC did not reverse this inhibition.

### PSC promotes osteoclast differentiation through the MAPK pathway

**Figure 5:**
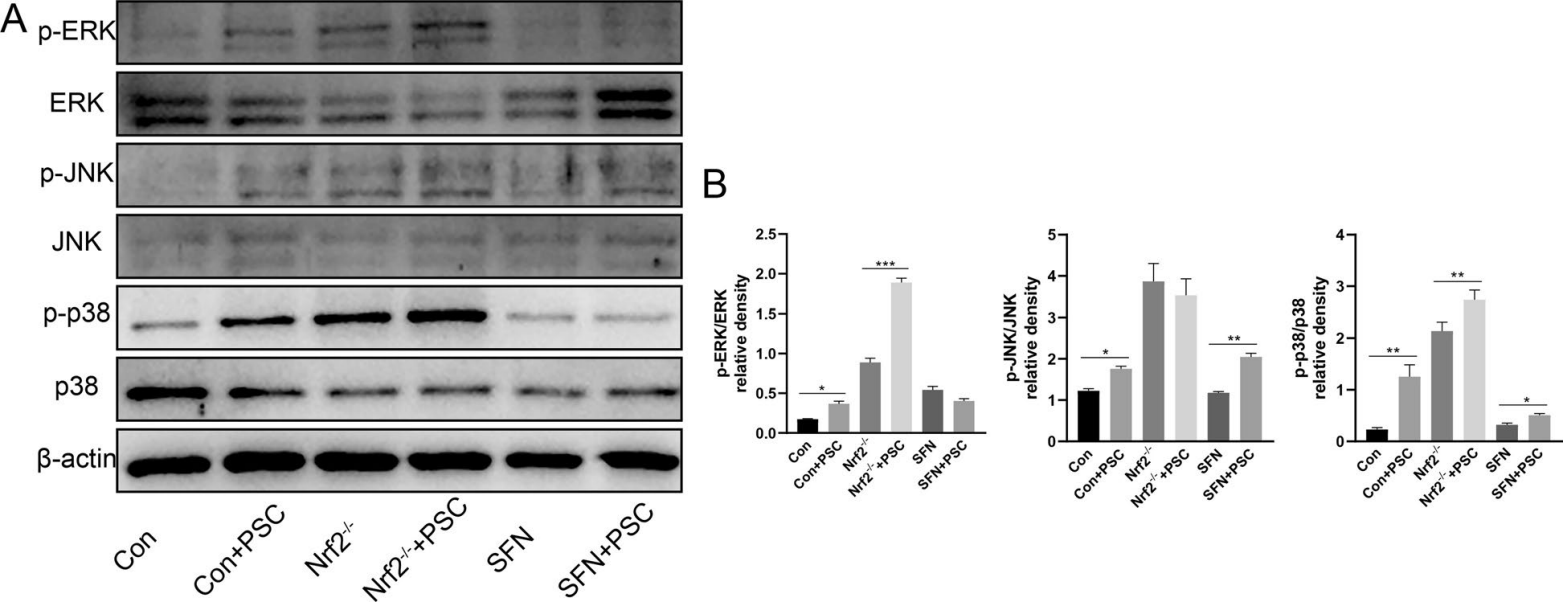
**Promotion of MAPK pathway activation after PSC intervention**. **A**, **B** Total protein was extracted after 3 days of culture in the normal induction, PSC induction, Nrf2 silencing, Nrf2 silencing with PSC induction, SFN, and SFN with PSC induction groups. Changes in the phosphorylation levels of the target proteins ERK, JNK, and p38 were examined by western blotting. *P < 0.05, **P < 0.01, ***P < 0.001.

The MAPK signalling pathway has also been widely studied as one of the core pathways regulating osteoclast differentiation and formation. Hyeon et al. reported that Nrf2^−/−^ mice presented increased osteoclast differentiation due to increased intracellular ROS levels, leading to the phosphorylation of p38 in the MAPK pathway. Activation of the MAPK pathway leads to increased activity of NFATc1, a key transcription factor for osteoclast differentiation, but not the NF-κB pathway [18]. Therefore, we examined the changes in the phosphorylation levels of ERK, JNK, and p38 in the MAPK pathway. Western blotting revealed that the phosphorylation of ERK, JNK, and p38 could be promoted to varying degrees by PSC intervention at different Nrf2 levels; the most significant promotion of MAPK pathway phosphorylation by PSC was observed when Nrf2 expression was silenced (Figures 5A and B). These results suggest that PSC can inhibit Nrf2 expression and thus promote MAPK pathway-mediated osteoclast differentiation.

### Nrf2 deficiency leads to more pronounced bone resorption in osseous CE

**Figure 6:**
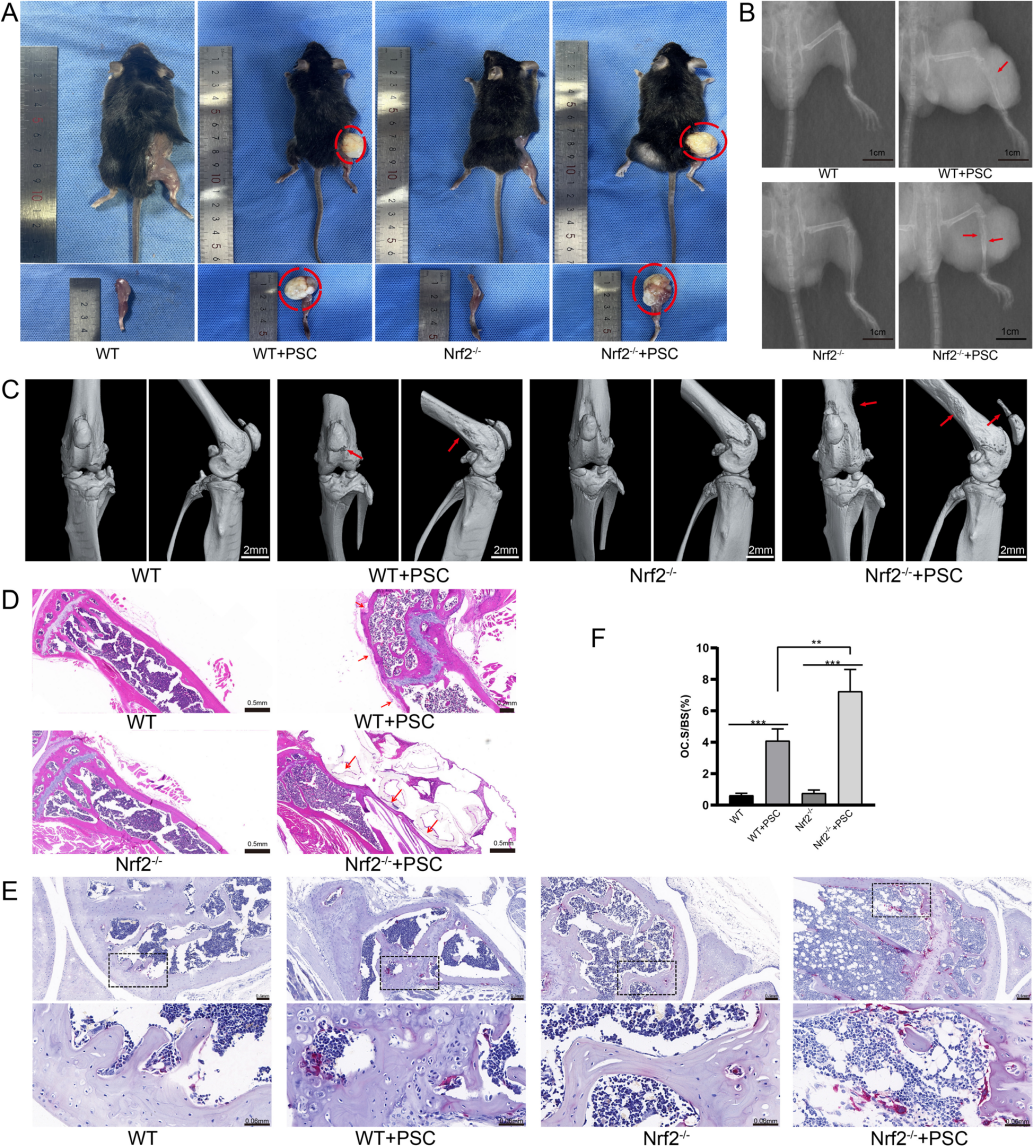
**Nrf2-deficient mice have more active bone destruction at the site of infection.** **A** Wild-type and Nrf2^−/−^ mouse femur CE models were established. **B** X-ray image showing destruction of bone cortical continuity after infection in a model group of mice. **C** Micro-CT scan of the femur in the CE model showed the presence of bone destruction. **D** Samples were collected for H&E staining after successful development of the model. **E**, **F** TRAP staining of bone tissue sections at the site of infection. The red clipped head indicates the site of bone destruction, and the black dotted box represents the magnified area. *P < 0.05, **P < 0.01, ***P < 0.001.

On the basis of the in vitro results, we further studied the related pathogenic mechanisms of osteoclastic activity in femoral CE in vivo. A disease model was constructed using wild-type and Nrf2^−/−^ C57BL/6 mice. Compared with those in the control group, both groups of mice in the model group formed vesicles in the lower limbs (Figure 6A). The infected groups of both types of mice exhibited varying degrees of bone destruction, as observed via X-ray and micro-CT scanning (Figures 6B and C). H&E staining of isolated tissues revealed that the bone cortex of the mice in the model group was significantly invaded by *E. granulosus* vesicles, with cortical bone thinning and bone trap formation. Bone destruction was more significant in the Nrf2^−/−^ mouse model than in the wild-type model (Figure 6D). Furthermore, TRAP staining revealed that the number of osteoclasts increased significantly after bone tissue infection, and the osteoclasts in the Nrf2^−/−^ mouse model were the most actively differentiated (Figures 6E and F). These results indicate that parasite-induced bone destruction was more pronounced and that bone resorption was more active in the absence of Nrf2.

## Discussion

Osteoclasts are the only cells known to possess bone resorption functions. In this study, we used a coculture system of PSC and BMMCs to demonstrate that PSC can promote osteoclast differentiation and active bone resorption. In our previous study, we showed that *E. granulosus* infection can lead to an increase in the differentiation of osteoclasts at the infected site and considerable bone loss [7]. While purely phenotypic studies cannot effectively validate clinical treatment, we reviewed the literature and found that, through coculture experiments and bioinformatics analysis, Wang et al. reported significant downregulation of the expression of the lncRNA ENSMUST00000132-226.1 [19], along with inhibition of the NF-κB signalling pathway via its target gene NLRP3 [20]. These findings may reveal the key mechanisms of bone destruction and provide a direction for future research. Researchers have identified genes that may be involved in the regulation of osteoclast differentiation through RNA bioinformatics analyses of PSCs [21]. These genes may encode key genes involved in the destruction of bone caused by *E. granulosus* infection. Therefore, the present study investigated Nrf2 after integrating the findings of several previous studies.

Numerous studies have demonstrated the important regulatory role of Nrf2 in osteoclast differentiation, with a particular focus on the effects of reactive oxygen species (ROS) and receptor activator of nuclear factor-kappa B ligand (RANKL) [22]. In osteoclasts, intracellular ROS are crucial for regulating differentiation processes [23, 24]. The binding of RANKL to its receptor, receptor activator of nuclear factor-kappa B (RANK), activates TRAF6, RAC1, and NADPH oxidase 1, resulting in increased intracellular ROS. ROS subsequently act as second messengers to activate downstream signalling pathways, including the MAPK, PI3K, and NF-κB pathways [25, 26]. Therefore, preosteoclasts must actively regulate their antioxidant response to maintain proper ROS levels and avoid excessive stress during osteoclastogenesis. When ROS production increases, Nrf2 is released from the Nrf2/Keap1 dimer into the cytoplasm. The released Nrf2 translocates to the nucleus, where it binds to antioxidant response elements (AREs), where it forms Nrf2-ARE heterodimers that promote the activation of antioxidant enzymes, such as heme oxygenase 1 (HO-1), to inhibit osteoclast differentiation [27]. Moreover, studies have shown that the expression of Nrf2 changes during *E. granulosus* infection and that the oxidative stress process is active in the early stage of infection, which leads to increased expression of Nrf2. However, *E. granulosus* infection is a long-term and chronic process, so chronic infection gradually inhibits the expression of Nrf2 and leads to the impairment of cellular antioxidant function [28, 29]. The dysregulation of antioxidant function leads to the gradual inhibition of Nrf2 expression [30]. Moreover, increased intracellular ROS activate the NF-κB and MAPK pathways, thereby promoting the differentiation and maturation of osteoclasts [31].

In our study, PSC intervention in BMMCs significantly suppressed the expression of Nrf2 as well as downstream effector HO-1 proteins and genes compared with that in controls, resulting in increased intracellular ROS levels. Reduced expression of Nrf2 was also observed in the femoral tissues of the *E. granulosus*-infected C57BL/6 mouse model. To further validate the regulatory role of Nrf2 in osseous CE disease, Nrf2^−/−^ BMMCs and the Nrf2 agonist SFN were used to modulate the intracellular Nrf2 levels in BMMCs. In vitro experiments revealed that osteoclast differentiation was promoted after the addition of PSC, and this effect was further amplified when Nrf2 was knocked down. Conversely, this promotional effect was significantly inhibited following Nrf2 activation. We also found that the level of intracellular ROS increased with PSC intervention, and this trend was positively correlated with osteoclast differentiation. Thus, the results of the in vitro experiments confirmed that PSC intervention led to the downregulation of Nrf2 and an increase in ROS levels in BMMCs, subsequently promoting osteoclast differentiation. For in vivo studies, an *E. granulosus*-infected femoral CE model was established using Nrf2 knockout mice, which exhibited more active osteoclast differentiation in bone tissues than normally infected mice without Nrf2 knockout.

The MAPK signalling pathway has been shown to play an important regulatory role in hydatid disease and macrophage function. Chong et al. used *E. multilocularis*-related antigen intervention in RAW264.7 cells and reported that it promoted M2 polarization of macrophages and activated the MAPK pathway, leading to liver fibrosis [32]. Lin et al. also demonstrated that the phosphorylation levels of the MAPK pathway-related proteins ERK, JNK, and p38 were significantly increased in *E. multilocularis* [33]. Li et al. reported high expression of p38 DNA Egp38 in *E. granulosus*, and in vitro experiments revealed that a p38 inhibitor (ML3403) significantly inhibited the activity of *E. granulosus* [34]. *E. granulosus* also expresses the MAPK-related molecules EgERK and EgMKK1/2, and inhibitors of these proteins (such as SB202190, sorafenib, and U0126-EtOH) significantly inhibit the activity of *E. granulosus* [35]. These studies demonstrate the potential for developing inhibitors targeting the MAPK pathway as specific anti-CE drugs.

Furthermore, changes in the phosphorylation levels of ERK, JNK, and p38 in the MAPK pathway under different Nrf2 expression conditions were verified. Western blotting revealed that the phosphorylation of ERK, JNK, and p38 was promoted to varying degrees after the addition of PSC at different Nrf2 levels. The phosphorylation of the MAPK pathway was most significantly promoted by PSC when Nrf2 expression was silenced.

In conclusion, our results elucidate the molecular regulatory mechanisms underlying increased osteoclast differentiation and active bone resorption in bone tissue at the site of *E. granulosus* infection, with Nrf2 playing a key regulatory role. Following *E. granulosus* infection, Nrf2 expression was significantly inhibited, and ROS levels increased, promoting the phosphorylation of the MAPK pathway, which ultimately led to an increase in osteoclast differentiation and bone erosion at the infection site. Understanding these molecular regulatory mechanisms can help target Nrf2, guiding more extensive research toward the ultimate goal of disease treatment. However, this study has several limitations, as the therapeutic effects of Nrf2 agonists and MAPK-related molecular inhibitors were not verified in the osseous CE mouse model, which will be the focus of subsequent research.

## Data Availability

The data that support the findings of this study are available in the Materials and Methods, Results, and/or Supplemental Material of this article.
